# Pregnancy and Lactation Associated Osteoporosis of the Hip: A Systematic Review

**DOI:** 10.1007/s00223-026-01493-y

**Published:** 2026-02-12

**Authors:** Madison L. Weckerly, Cailan L. Feingold, Naitik K. Singh, Mary K. Fatehi, Robert Abrahams, Daniel A. Mirzai, Adi Cohen, Anna Cooper

**Affiliations:** 1 New York Medical College, New York, USA; 2 Columbia University, New York, USA; 3 Montefiore Einstein, New York, USA

## Abstract

During pregnancy and lactation, changes in mineral metabolism provide fetuses with sufficient calcium. Eith a reported instance of 4–400 per 1 million pregnancies, the decrease in bone mineral density causes pregnancy and lactation associated osteoporosis (PLO). This the first systematic review conducted on this condition affecting the hip. A comprehensive literature search of the databases Embase, PubMed, and Web of Science was conducted. The articles included for review were case studies or case series that consisted of pregnant patients with PLO of the hip. Data extraction was performed on the resulting 64 studies, totaling 149 patient cases. The average age reported was 33.5 years, with an average BMI of 26.5 kg/m^2^ and an average height of 165.8 cm. Symptom onset was reported in 50% of cases, 69% in the 3rd trimester, 25% in the 2nd trimester, and 4% in the postpartum period. The route of delivery was a cesarean section in 44% of cases, vaginal delivery in 33%, and unreported in 23%. Treatment included modified weight-bearing (40%), vitamin D and/or calcitriol (26%), and surgery (26%). Cesarean section in patients with PLO occurred at a greater percentage (44%) when compared to the rate of cesarean section in the United States in 2023 (32.1%). This could have implications for the health of the mother and the fetus. The majority of cases occurred during pregnancy. This may complicate the diagnosis and care of the patient. The absence of metabolic disease workup details in these cases is an area for future research and clinical focus.

## Highlights

Pregnancy and Lactation Associated Osteoporosis

Pregnancy

Osteoporosis

## Introduction

During pregnancy and lactation, the mineral metabolism of women undergoes changes to provide sufficient calcium availability to the growing fetus or infant. The majority of the increase in calcium during pregnancy results from a doubling in calcitriol levels, which greatly increases the absorption of calcium from the gastrointestinal tract [1, 2]. In contrast, parathyroid hormone-related protein (PTHrp) released by breast tissue stimulates osteoclast-driven bone resorption from the maternal skeleton during the lactation period to provide sufficient calcium in the breast milk [1, 3, 4]. Normally, the decreasing PTHrp and rising estradiol levels, which occur with the cessation of lactation and the resumption of menses, results in the restoration of the lost bone mineral density (BMD) which most studies indicate occurs by 12 months post-weaning [1, 5].

In the majority of cases, these metabolic changes are clinically silent; however in a reported instance of 4–460 per 1 million pregnancies, fractures occur in the context of these changes – a condition called pregnancy and lactation associated osteoporosis (PLO) [1, 2, 5–7]. Some studies and reviews define PLO as a condition resulting in vertebral fractures, while others include presentations of the condition with fractures of the hip, pelvis, sacrum, ribs, and extremities [8–11]. Patients typically present during the lactation period with an average of 4–5 vertebral fractures, and second most commonly during pregnancy with an affected proximal femur [1, 7]. With a lower occurrence rate, PLO of the hip has been studied less frequently, with most research available largely consisting of case studies and case series.

When affecting the hip, PLO has historically been referred to as “transient osteoporosis of the hip” (TOH), and has been identified in pregnant patients in the literature dating back to 1959 [12]. TOH was defined as pain in the hip that resolved with or without evidence of osteopenia on radiograph and could occur in pregnant women along with nonpregnant women and men [1]. In contrast, diagnosis of PLO requires a pregnant or postpartum patient along with evidence of bone injury, which includes bone marrow edema found on magnetic resonance imaging (MRI) or cortical defects such as stress, insufficiency, or full thickness-fractures found on any imaging modality. True distinction between these two etiologies of hip pain is still being investigated, including genetic contributions to the pathogenesis of PLO. Recent research has identified heterozygous rare predicted deleterious variants in genes related to the WNT bone formation pathway in patients with both vertebral and hip fracture presentations [13]. In most of the currently available case studies and series of this disease affecting the hip, genetic investigation has not been conducted, and distinguishment between TOH and PLO is often not discussed. Differences between these diseases are being elucidated with continuing research [1]. TOH has also been likened to conditions including avascular necrosis of the hip (AVN), however more recent studies of men and women with TOH understand the radiographic finding of intense bone marrow edema on MRI to represent insufficiency fractures of the hip, distinguishing the condition from AVN [14]. The pathophysiology of PLO of the hip, in comparison to the spine, is more poorly understood with bone resorption, ischemia, and pressure from the gravid uterus possibly contributing [5].

Available studies provide inconsistent evidence regarding the bone loss that occurs during pregnancy, while more are conclusive that significant bone loss occurs during lactation [5]. Moller et al. found a significant decrease in the bone mineral density of healthy pregnant patients when compared to nonpregnant patients, with adjustments made for changes in body weight and composition. They found an average BMD loss of 1.8 ± 0.5% at the lumbar spine, 3.2 ± 0.5% at the hip, and 2.4 ± 0.3% at the whole body compared to the nonpregnant group [15]. The ability to conduct studies measuring BMD in pregnant patients is limited in order to limit fetal exposure to radiation, with MRI’s predominating as the diagnostic method of choice [16].

As a rarely diagnosed condition occurring in a vulnerable patient population, available research is limited [11]. A systematic review has been conducted studying vertebral involvement in PLO but this is the first systematic review conducted on this condition affecting the hip [17]. This study aimed to gather and present all available data reported in case studies and case reports of this condition.

## Methods

### Literature Search

**Fig. 1:**
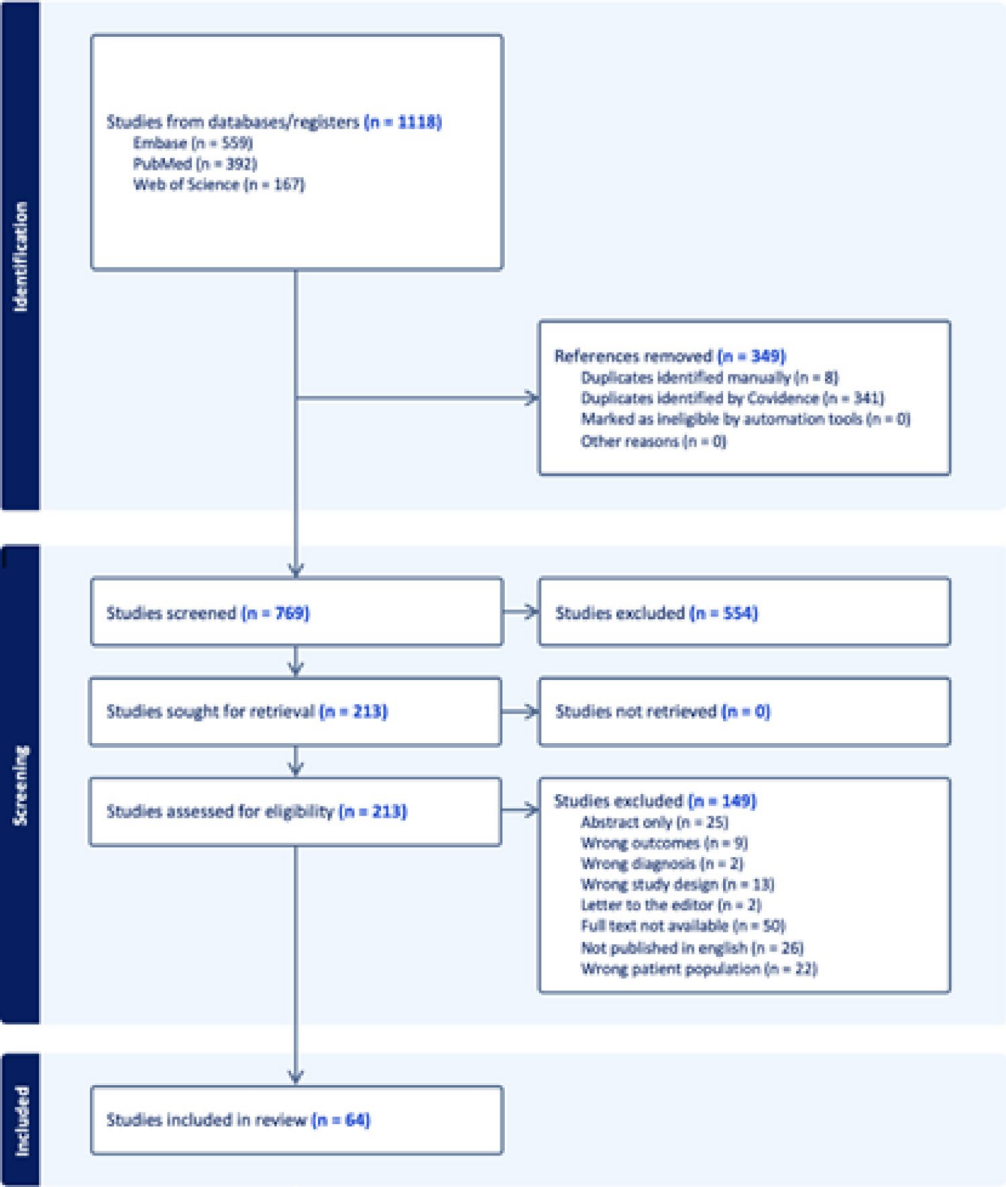
Flow diagram of study inclusion and exclusion

A comprehensive literature search of PubMed, Embase, and Web of Science was conducted using the terms "pregnancy associated osteoporosis” OR “lactation associated osteoporosis” OR "transient osteoporosis" OR "pregnancy and lactation associated osteoporosis" OR “pregnancy” OR “lactation” OR “postpartum” AND "intertrochanteric fracture” OR "femur fracture" OR "femoral neck fracture" OR "hip fracture" OR "hip" OR "femur”. When querying these three databases, years 1987 through 2024 were assessed. Studies were compiled in Covidence (Melbourne, Australia) and 349 duplicates were removed (Fig. 1).

### Screening

Studies were included if they were case reports and/or case series reporting on patients that developed PLO of the hip during or after pregnancy. Studies had to be published in English and peer-reviewed journals. Excluded studies were literature reviews, reports of non-pregnant patients, patients with PLO or fracture of other anatomic regions, and patients with a different diagnosis of the hip such as avascular necrosis. If a study was a case series that reported on multiple patients, only pregnant patients with PLO of the hip were collected. Cases were included for analysis only if a description of bone injury was present, including a finding of bone marrow edema on MRI or cortical defect present on imaging. Two authors, M.W. and C.F. independently screened studies. Screening was done in a two-phase process, first by title and abstract, then by full text. If the studies’ full texts were inaccessible, they were excluded.

### Data Collection

Patient demographics including age, body mass index (BMI), and height were collected independently by two authors, M.W. and N.S. Onset of symptoms by trimester and route of delivery were collected when available. Type of diagnostics used, and findings of injuries were recorded, as well. Treatment and management tools utilized were collected. Any patient habits or other medical history reported were recorded, including but not limited to history of substance use and prior obstetric history. Data was recorded descriptively in Excel (Version 16.94, Microsoft, Tacoma WA).

Individual data elements were extracted when reported. For continuous variables, summary measures were calculated using only cases in which the variable was explicitly reported. No imputation was performed for missing data. For categorical variables, two complementary proportions were calculated. First, the reporting frequency was determined by calculating the percentage of included studies that provided the specific data element. Second, among cases in which the data element was reported, proportions were calculated to describe the distribution of outcomes. All percentages were therefore denominator-specific and reflect either (1) the proportion of cases with available data or (2) the proportion of outcomes among cases with reported data, as explicitly stated for each analysis. Due to the descriptive nature of the included evidence, formal meta-analysis and pooled effect estimates were not performed.

### Quality Assessment

Two reviewers independently assessed the quality of the included studies and disagreements were solved through discussion among authors. The Joanna Briggs Institute (JBI) critical appraisal checklist for case reports and case series were used to evaluate the quality of all included studies [18]. There are eight questions in the checklist for case reports and ten questions in the checklist for case series. The reviewers decided that the studies that graded as a “yes” in a minimum of 50% of the questions achieved adequate quality for inclusion.

## Results

Data extraction was performed on the resulting 64 studies, totaling 149 patient cases. Age was the most reported statistic in cases of PLO, with an average of 33.5 years (SD = 3.9). BMI and height were reported in 60% and 56% of cases respectively and had averages of 26.5 kg/m^2^ (SD = 7.7) and 165.8 cm (SD = 3.9). Symptom onset was reported in 50% of cases, 69% of which were in the 3rd trimester, 25% in the 2nd trimester, and 4% in the postpartum period. Of note, Toussia Cohen et al. reported 50% of their cases were diagnosed in pregnancy and the other 50% were diagnosed postpartum, but the timing of onset of symptoms was not reported, as a result the participants were not included in the analysis of onset of symptoms. The gravida of patients was the first pregnancy in 35% of cases, 19% in the second, and 11% in the third, and one case was in their fifth, with 34% of cases of unreported gravida. The route of delivery was a cesarean section (CS) in 44% of cases, vaginal delivery in 33%, and unreported in 23%.

Symptoms reported throughout the duration of a case report were included in this analysis. The most common symptom was hip pain in 74% of cases, followed by an alteration in weight-bearing in 31%, decreased range of motion in 17%, knee pain in 7%, and low back pain in 5% of cases. Hadji et al. noted immobility and restricted movements in their participants but didn’t provide individual data and could not be included in our analysis (Table 1).

**Table 1:** Study characteristics

Author	Year published	Number of cases	Age	Height (cm)	BMI (kg/m^2^)	Type of delivery
Soares	2024	1	39			Cesarean
Thanasa	2024	1	31		32	Cesarean
Toussia-Cohen	2023	34	34.18	165.25	27.6	Cesarean 16 Vaginal 16 N/A 2
Bhakta	2022	1	40		28	Cesarean
Factor	2022	1	38			Cesarean
Siva	2022	1	35			Cesarean
Al-Dourobi	2021	1	24		40	Vaginal
Klimko	2021	1	38			Cesarean
Philip	2021	1	26	153.1	32	Cesarean
Quaresima	2021	1	32		26	Cesarean
Scioscia	2021	1	36			
Wright	2021	1	24		32.8	Cesarean
Faraji	2020	1	29		22	Cesarean
Kha	2020	1	33			Cesarean
Carriles Rivero	2019	1	36			Cesarean
Jun Jie	2019	1	33			Vaginal
Paiva	2019	1	48			Cesarean
Sahan	2019	1	28	165	31.2	Cesarean
Tayne	2019	1	32			Cesarean
Kasahara	2018	1	40	161	15.4	Cesarean
Hadji	2017	33	35.2	167.5	23.8	Cesarean 12 Vaginal 19 N/A 2
Kasahara	2017	1	38	157	16.2	Cesarean
Thanatsis	2017	1	37			Cesarean
Lidder	2015	1	31			Vaginal
Reese	2015	3	41			Cesarean
Scapinelli	2015	2	33	167.5	21.75	Cesarean 1 Vaginal 1
Anai	2013	1	32			Cesarean
Uzun	2013	1	27			Vaginal
Vester	2013	2	33			
Jafar Emami	2012	1	36			Cesarean
Pallavi	2012	1	30			Cesarean
Patel	2012	1	34	160	23.91	Cesarean
Truszczynska	2012	2		163	20.3	Cesarean
Bin Abdulhak	2011	1	28			Cesarean
Kim	2010	1	28			
Chalouhi	2009	1	30			Cesarean
Lamarca	2009	1	29	172	33.5	Vaginal
Spinarelli	2009	1	35			
Diwanji	2008	2	38.5			
Willis-Owen	2008	1	34			Cesarean
Xyda	2008	3	31			
Aynaci	2007	1	23	167	20.5	Cesarean
Cohen	2007	1	37			Vaginal
Steib-Furno	2007	6	31.5			
Montagna	2005	1	36			Cesarean
Bezer	2004	3	28.3			
Arayssi	2003	2	37			
Wood	2003	1	29			Vaginal
Fazekas	2002	1	38			
Axt-Fliedner	2001	1	35			Cesarean
Boissonnault	2001	1	32			
Sweeney	2000	1	28	173	24.3	
Thomas	2000	1	26			Vaginal
Uematsu	2000	4	31.8			
Yamamoto	1999	1	33			
Fokter	1997	1	30			Cesarean
Jolliffe	1997	1	27			Cesarean
Siva	1997	1	31	157	24.3	Cesarean
Junk	1996	1	35			
Fingeroth	1995	1	27	146	90	Cesarean
Funk	1995	1	31	180	22.1	Vaginal
Goldman	1994	1	34			Vaginal
Brodell	1989	2	31			Vaginal
Shifrin	1987	1	23			Vaginal

Imaging modalities used throughout the cases included MRI, x-ray, and CT. MRI was the most used modality as evidenced in Table 2. X-rays were the second most frequently used modality, and CT was only obtained in 6% of cases. Dual-energy x-ray absorptiometry (DEXA) scans were completed in 17% of cases, but scores were not consistently reported, and analysis could not be performed.

**Table 2:** Imaging

Imaging and findings	Number of cases	Percentage of cases (%)
*MRI*		
Obtained	130	87
Edema	126	85
Fracture	2	1
*Xray*		
Obtained	57	38
Fracture	30	20
Demineralization/osteopenia	29	19
Normal	4	3
*CT*		
Obtained	9	6
Fracture	5	3
Demineralization/Osteopenia	4	3

The “Number of Cases” column describes of the 149 patient cases, in how many of the cases was the imaging obtained

The “Number of Cases” column also describes how many of the 149 cases had the findings of “Edema”, “Fracture”, “Demineralization/Osteopenia”, or “Normal”

Cortical defects described as fractures occurred in 41 cases, with a total of 51 fractures occurring due to bilaterality occurring in 10 cases. Fracture site and laterality is further described in Table 3. Of the 41 cases in which fractures were reported, 38 (93%) of those patients were treated with surgical fixation of their hip. All other reported treatments, including their frequencies, has been noted in Table 4

**Table 3:** Fracture specification

Fractures	Number of cases	Percentage of cases (%)
Femoral neck fracture	36	26
**Subcapital specifically**	**19 of 36**	**53 of 71**
Femoral Head	4	10
Acetabulum	2	2
Inter trochanteric	1	2
Unspecified	7	14
Sacral	1	2
Total fractures	51	
Bilateral	10	24
Right	11	27
Left	12	29
Unspecified/not applicable	8	20
Total cases with fractures	41	28

The “Number of Cases” column reports of the 149 total cases analyzed, how many had each type of fracture and how many reported each laterality

The percentages are reflective of the number of cases per the 149 total cases

Fractures may have been diagnosed on MRI, X-ray, or CT

Because of the occurrence of bilateral fractures, there are more Total Fractures reported then there are Total Cases with Fractures

**Table 4:** Treatments

Treatments	Number of cases	Percentage of cases (%)
Surgery	38	26
Bisphosphonates	8	5
Calcitonin	6	4
Calcium supplements	32	21
Vit. D/calcitriol	39	26
Modified weight bearing	59	40
Bed rest	24	16
NSAIDs/analgesics	55	37
Physical therapy	45	30
Traction	3	2
Anticoagulation	18	12
Unspecified	38	26
Teriparatide	3	2

The “Number of Cases” column describes of the 149 patient cases, in how many of the cases was that treatment modality reported

The percentages are reflective of the number of cases per the 149 total cases

Risk factors noted include tobacco use in 15% of cases, a history of abortion or miscarriage in 3%, an eating disorder in 2%, and gestational diabetes in 3%. A history of coagulation disorders, thyroid disorders, and osteoporosis were each reported in 1% of cases. Hadji et al. found associations between PLO and dental problems in childhood, exercise before and after puberty, and weight gain during pregnancy. Toussia-Cohen et al. also noted dental disease above normal in 26% of their cases.

## Discussion

In this study, we performed a systematic review of PLO of the hip and analyzed multiple factors, including prevalence of risk factors, rates of cesarean section compared to vaginal delivery, the methodology of diagnosis, and treatments utilized. Due to a lack of consistency across the case studies and series limited the assessment of various characteristics of each case. Risk factors is one of the categories in which this was the most evident. Current literature describes risk factors including a low BMI (< 18 kg/m^2^), immobility, dental problems, a history of an eating disorder, smoking, and low vitamin D levels as potential risk factors [4, 5, 19]. No cases analyzed in our review described a presence or lack of all postulated factors. Tobacco use in 15% of cases was the most reported risk factor, followed by a history of abortion or miscarriage in 3%. The average BMI 26.5 kg/m^2^, but it is uncertain during which point in their pregnancies each case’s BMI was measured, making it difficult to compare it to an average or ideal. Dental disease was noted in two large case series included in this study, Hadji et al. and Toussia-Cohen et al. as well as an association between decreased exercise levels before and after puberty and PLO by Hadji et al. [20, 21].

In cases with a reported route of delivery, CS occurred at a greater percentage (44%) when compared to the US population in 2023 (32.4%) [22]. Some cases described hip fractures occurring as a result of a vaginal birth, substantiating a potential greater need for CS in PLO of the hip. While CS could reduce rates of fracture or mitigate complications of already occurring fractures, the benefits and risks of CS should still be taken into consideration, including an estimated four times higher risk of maternal death compared to vaginal deliveries, as well as an increased chance of obstetric shock, hysterectomy, and placental abruption in future pregnancies [23, 24].

The onset of symptoms occurred during pregnancy in 94% of cases. This may complicate the diagnosis and care of the patient in an attempt to reduce fetal exposure to imaging modalities and treatments with known or unknown teratogenic effects. Ultrasound and MRI are not associated with an increased risk in the mother or fetus, while imaging modalities utilizing ionizing radiation are associated with risks but should not be withheld if decided it’s clinically necessary [25–27]. MRI is the most used imaging modality in the literature for this condition, with a characteristic finding of bone marrow edema confirming the diagnosis [5, 16]. MRI findings were by and far the most reported diagnostic modality reported in this review as noted in Table 2, with consistent findings of bone marrow edema reported.

Treatment of PLO of the hip is controversial as BMD is expected to be regained in 6–12 months after the cessation of lactation [2, 3]. In addition, osteoporotic medications such as bisphosphonates, denosumab, and teriparatide, are contraindicated during pregnancy and breastfeeding. Vitamin D and calcium, the most prescribed medications given in this analysis at 26% and 21% of cases respectively, are the current recommended supplements to take during pregnancy and lactation, as both are safe during pregnancy [5, 19, 28–30]. No randomized control trials have been performed to assess the efficacy or safety of osteoporotic medications, but bisphosphonates, denosumab, and teriparatide are reported in case studies and series as prescribed treatments, as seen in our analysis. It’s also important to note that of the 41 patients described as having fractures, 93% of those patients underwent surgical fixation of their hip. Further research is needed to determine a consensus on treatment for PLO.

Emerging evidence suggests that genetic factors contribute meaningfully to the pathophysiology of PLO. In a recent study, Lynch et al. identified potentially pathogenic genetic variants in approximately 30% of affected patients, including variants in *WNT1*, *LRP5*, and *LRP6*, as well as genes involved in calcium homeostasis, supporting a substantial genetic predisposition in a subset of cases [13]. Inherited disorders of bone fragility may be unrecognized contributors to PLO in a subset of reported cases. Monogenic conditions, including mild or late-onset forms of osteogenesis imperfecta and variants affecting the WNT signaling pathway (e.g., *LRP5* and *WNT1*), have been increasingly recognized as causes of primary osteoporosis and may be unmasked during periods of increased skeletal demand such as pregnancy and lactation [31, 32]. Notably, none of the case reports or series included in this review documented formal genetic evaluation or testing, limiting the ability to exclude underlying genetic bone disorders and representing an important limitation of the current literature [33].

## Conclusion

PLO of the hip is a rare but clinically significant condition that remains poorly understood, with limited available data primarily derived from case reports and series. The variability in reporting of patient histories and clinical characteristics complicates comprehensive analysis, though factors such as smoking and dental disease may contribute to disease susceptibility. Diagnosis is further challenged by the need to minimize fetal exposure to ionizing radiation, with MRI remaining as the preferred imaging modality. There are potential associations with poor maternal and fetal outcomes with PLO, including preterm births and delivery complications. Given the functional and obstetric implications of PLO, future research is necessary to establish standardized diagnostic criteria, risk stratification, and management strategies to optimize maternal and fetal outcomes.
